# The prognostic significance of CXCL1 hypersecretion by human colorectal cancer epithelia and myofibroblasts

**DOI:** 10.1186/s12967-015-0555-4

**Published:** 2015-06-24

**Authors:** Anne-France le Rolle, Thang K Chiu, Michael Fara, Jinru Shia, Zhaoshi Zeng, Martin R Weiser, Philip B Paty, Vi K Chiu

**Affiliations:** Division of Hematology/Oncology and Chao Family Comprehensive Cancer Center, Department of Medicine, University of California, 839 Health Sciences Road, Sprague Hall Office 116, Irvine, CA 92697 USA; Department of Biochemistry and Molecular Biology, Louisiana State University Health Sciences Center, New Orleans, LA 70112 USA; Department of Medicine, Weill Cornell Medical College, New York, NY 10065 USA; Department of Pathology, Memorial Sloan-Kettering Cancer Center, New York, NY 10065 USA; Department of Surgery, Memorial Sloan-Kettering Cancer Center, New York, NY 10065 USA

**Keywords:** CXCL1, Chemokine, CRC prognosis, KRAS, Tumor microenvironment

## Abstract

**Background:**

Clinical therapy for metastatic colorectal cancer (CRC) remains limited, especially when the tumor harbors a KRAS mutation. This study aimed to identify prognostic biomarkers in CRC that are accessible for therapeutic inhibition.

**Methods:**

Conditioned media from human CRC epithelial cells and myofibroblasts were screened by cytokine arrays for tumorigenic factors. The protein and mRNA expressions of these factors were determined by immunohistochemistry and gene microarrays in human CRC tissues. Prognostic biomarkers were determined by correlation of mRNA expression to overall survival in stage IV CRC patients. Inhibition of CXCL1 was performed with specific neutralizing antibody and lentiviral shRNAs. Malignant growth was assessed by soft agar growth assays and xenograft tumor growth in immunocompromised mice.

**Results:**

CXCL1 was highly secreted by KRAS mutant human CRC cells and myofibroblasts in a complementary adaptive response to serum deprivation. Elevated CXCL1 level promoted anchorage-independent growth of murine fibroblasts and human CRC cells. Inhibition of CXCL1 by neutralizing antibody and specific shRNAs decreased CRC tumor growth. Highly elevated CXCL1 expression significantly correlated with decreased overall survival in stage IV CRC patients (hazard ratio 0.28; 95% CI 0.11–0.72).

**Conclusions:**

High CXCL1 expression is a poor prognostic biomarker in metastatic CRC. CXCL1 inhibition suppressed tumorigenic growth of KRAS mutant CRC cells.

**Electronic supplementary material:**

The online version of this article (doi:10.1186/s12967-015-0555-4) contains supplementary material, which is available to authorized users.

## Background

Colorectal rectal cancer (CRC) is the third most commonly diagnosed cancer globally with over 1.2 million new occurrences annually [1]. The pathogenesis of CRC involves a complex and adaptive tumor microenvironment consisting of malignant epithelia with surrounding stromal cells such as myofibroblasts and inflammatory cells [2]. Autonomous and sustained mitogenic signaling in a nutrient poor environment is a fundamental hallmark of cancer [3, 4]. Crosstalk between tumor epithelia and stroma has emerged to have active roles in tumorigenicity. Both activated and mutated fibroblasts may induce autocrine and paracrine factors that mediate epithelia to adenocarcinoma transformation [5, 6]. In a human breast cancer model, co-culturing of mesenchymal stem cells with breast cancer cells enhances invasiveness through increased chemokine signaling [7]. Notably, gene expression profiles of microdissected human breast cancer stroma have high prognostic significance, with angiogenesis and immune-related gene clusters associated with poor and favorable prognoses, respectively [8]. In human CRC, myofibroblasts are also implicated in tumor invasion [9]. Indeed, an increased presence of α-smooth muscle actin expressing myofibroblasts in resected stage II–III CRC was an indicator of higher cancer recurrence and poor prognosis [10]. The specific interactions within human colon cancer epithelia and stroma that significantly impact on overall survival remain unclear.

The principal therapy for metastatic CRC therapy relies on direct killing of the tumor with cytotoxic chemotherapy. Recent gains in overall survival have been achieved with extracellular targeting of the tumor and its microenvironment, namely bevacizumab inhibition of angiogenesis, cetuximab or panitumumab inhibition of EGFR signaling by EGF family ligands, and regorafenib inhibition of multiple targets [11–14]. Secreted cytokines, chemokines and acute phase reactants in the tumor microenvironment represent attractive and readily targetable biomarkers if they are major drivers of tumor progression in humans [15]. To increase our understanding of therapeutically relevant secreted factors within the human CRC tumor microenvironment, we surveyed the conditioned media of human CRC epithelial cells and myofibroblasts for tumorigenic factors that remain elevated under serum nutrient deprivation. As these tumorigenic factors may promote tumor survival, we determined the extent that their in vivo expression levels in human primary CRC tissues impact overall survival. We have identified CXCL1 as a constitutively expressed tumorigenic factor whose excess production is associated with a significant decrease in overall survival of stage IV CRC patients.

## Methods

### Human mucosal tissues

Human tissues were collected prospectively under an IRB approved protocol from patients undergoing elective surgery for CRC at Memorial Sloan-Kettering Cancer Center from January 1990 to December 2000. Tissues included normal colon, normal liver, colorectal adenomas, primary CRC, and liver and lung metastases from primary CRC. Mucosal tissues were pathologically verified and obtained by manual microdissection under microscopic visualization to remove adjacent connective tissue and muscle.

### Cells and reagents

Murine NIH3T3 (wildtype KRAS) and human CRC cells SW48 (wildtype KRAS) and SW620 (mutant KRAS) were obtained from American Type Culture Collection (ATCC; Manassas, VA, USA). Human CRC myofibroblasts cells (CRC-MF) were isolated from a primary human colorectal liver metastasis under an IRB approved protocol and expressed α-smooth muscle actin. All cells were cultured in Dulbecco’s Modified Eagle Medium (DMEM) from Invitrogen (Carlsbad, CA, USA) supplemented with 10% fetal bovine serum and penicillin, and streptomycin. Antibodies were obtained from R&D Systems (goat anti-human CXCL1 antibody, anti-human IL8 antibody, and control isotypic IgG antibody), Invitrogen (donkey anti-goat IgG-Alexa Fluor® 568 conjugate antibody) and Sigma (mouse anti-α-smooth muscle actin-FITC clone 1A4 antibody).

### Generation of lentiviruses

EGFP and neomycin resistant genes were cloned into pCCL-PGK lentivirus vector (pCCL-PGK-GFP). KRAS12V ORF was amplified by PCR from pcDNA3.1^+^neo-Kras12V using forward primer 5′-GGG GGA TCC ACC GCC ATG ACT GAA TAT AAA CTT GTG-3′ and reverse primer 5′-GATT GTC GAC TTA CAT AAT TAC ACA CTT TGT CTT TGA C-3′ and cloned into the 5′BamHI and 3′SalI sites of pCCL-PGK (pCCL-PGK-KRAS12V). CXCL1 and non-target control lentiviral shRNA TRC1 vectors were obtained from Sigma-Aldrich. Lentiviruses were generated by cotransfecting 15 µg of lentiviral vector (pCCL-PGK-GFP or -Kras12V), 3.5 µg of pENV/VSV-G, 6 µg of pRRE, and 3 µg of pRSV-REV into 293T cells in 10 cm plates using the calcium precipitation method. Lentiviral supernatants were collected at 40 and 64 h after transfection. Cells were transduced with lentiviruses at MOI = 10 and maintained in DMEM growth medium.

### Conditioned media

Sixteen millions cells of SW620 and CRC-MF were cultured in 20 ml of DMEM supplemented with 10% fetal bovine serum. On day 3, the supernatants were collected and the adherent cells were washed twice with PBS and maintained in serum-free DMEM for an additional 3 days. Conditioned media were collected from day 3 serum^+^ supernatants and day 6 serum-free supernatants, centrifuged for 10 min at 400 g to remove cellular debris, sterile filtered through 0.22 µm Millipore filter (Billerica, MA, USA), and stored at −80°C prior to use.

### Soft agar growth assay

Ten thousand NIH3T3 cells and one thousand SW620 cells were grown in a 0.33% top layer and a 0.5% bottom layer that included Difco Bacto Agar (w/v). The top layer contained growth media or conditioned media and the bottom layer contained only growth media. On day 14, colonies greater than 50 cells were counted visually using a stereo zoom microscope. Neutralizing anti-human CXCL1 monoclonal antibody, anti-human IL8 monoclonal antibody, and control isotypic antibody (R&D Systems) at 625 ng/ml of total volume were added at the initiation of soft agar assays.

### Murine xenograft tumor assay

Four million human colon cancer cells were injected into the dorsal subcutaneous flank of *Foxn1nu/Foxn1nu* nude mice. Growth of tumor xenografts were measured with a caliber at the indicated time. All mouse experiments were done in compliance with the Institutional Animal Care and Use Committee (IACUC) policies.

### Elisa

Conditioned media were evaluated for the relative presence of 57 different cytokines, chemokines, and acute phase reactants using the Human Cytokine Array Kit, Panel A and Human Chemokine Array from R&D Systems (Minneapolis, MN, USA) according to manufacturer’s instructions. These included CCL1/I-309, CCL2/MCP-1, CCL3/MIP-1a, CCL4/MIP-1b, CCL5/RANTES, CCL7/MCP-3, CCL14/HCC-1/HCC-3, CCL15/MIP-1d/LKN-1, CCL17/TARC, CCL18/PARC, CCL19/MIP-3b, CCL20/MIP-3a, CCL21/6Ckine, CCL22/MDC, CCL26/Eotaxin-3, CCL28, Chemerin, C5a, CD40 ligand, G-CSF, GM-CSF, CXCL1/GRO alpha, CX3CL1/Fractalkine, CXCL4/PF4, CXCL5/ENA-78, CXCL7/NAP-2, CXCL8/IL-8, CXCL9/MIG, CXCL10/IP-10, CXCL11/I-TAC, CXCL12/SDF-1, CXCL16, CXCL17/VCC-1, ICAM1, IFNg, IL1a, IL1b, IL1RN, IL2, IL4, IL5, IL6, IL10, IL12, IL13, IL16, IL17, IL17E, IL23, IL27, IL32 alpha, Lymphotactin/XCL1, Midkine, MIF, Serpin E1, TNF-alpha and TREM1. Signal intensities were determined from measuring pixel intensity (PI) of scanned images and quantified with Adobe Photoshop (San Jose, CA, USA). Briefly, conditioned media were diluted, mixed with a cocktail of biotinylated detection antibodies and incubated with the cognate immobilized capture antibody on the cytokine array membrane. Streptavidin-horseradish peroxidase and chemiluminescent detection reagents were added and a signal was produced in proportion to the amount of cytokine bound. The cytokine array membranes were scanned as TIF images and the signal minus background were quantified as pixel intensities (PI).

Quantitative levels of CXCL1 and IL8 in the conditioned media were measured using CXCL1 and IL8 Quantikine Assay kits (R&D Systems) according to manufacturer’s instructions. The measured optical densities of CXCL1 and IL8 in conditioned media were converted to concentrations (ng/ml) by linear regression analysis of optical densities and standard concentrations of recombinant CXCL1 and IL8 proteins.

### Immunofluorescence

Formalin fixed paraffin embedded human CRC were sectioned at 4 µm. CRC tissue paraffin slides were warmed for 30 min at 60°, deparaffinized in xylenes and graded alcohols, treated with antigen retrieval buffer (DAKO) in a steamer for 30 min, washed in PBS and blocked for 1 h at room temperature with 10% Normal Donkey Serum and 0.02% Tween-20 in PBS. Primary goat anti-human CXCL1 antibody and mouse anti-α-smooth muscle actin-FITC clone 1A4 antibody were incubated overnight at 4° and washed away with PBS prior to incubation for 1 h at room temperature with secondary donkey anti-goat IgG-Alexa Fluor® 568 conjugate antibody. Labeled tissues were sealed with glass covers with Cytoseal and immunofluorescence images were collected with Zeiss LSM 780 confocal microscopy.

### Expression profile of human mucosal tissues

Total RNA was purified with Qiagen RNeasy purification column and reagents (Invitrogen) from human mucosal tissues. After reverse transcription, the cDNA was hybridized to Affymetrix Human Genome U133A (mucosal tissues) or U133 Plus 2.0 (SW48 cells) GeneChips per manufacture protocol. MAS-3 software was used for obtaining raw signal intensity (SI) data from the gene chips. CXCL1 and IL8 mRNA expression values >3 standard deviations above the mean of baseline normal control were considered as significantly gained above normal.

### Statistics

Statistical analyses for mean, standard error of the mean, Spearman correlation, Log-rank test for Kaplan–Meier survival curves and linear regression analysis for CXCL1 and IL8 levels, were performed with GraphPad Prism v5.0d (San Diego, CA, USA). Statistical significance for multi-groups comparison was based ANOVA.

## Results

### Human colorectal epithelial cells and myofibroblasts secrete tumorigenic factors

**Figure 1:**
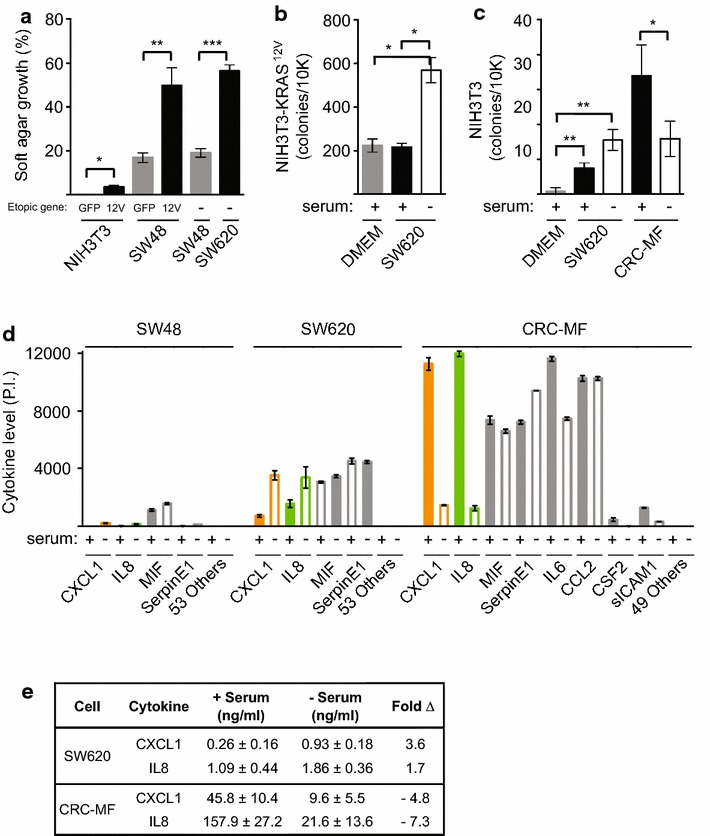
Conditioned media of human CRC epithelial cells and CRC myofibroblasts induced anchorage-independent growth. **a** Percent efficiency of anchorage independent growth in soft agar assays of murine NIH3T3 cells that overexpressed GFP or KRAS^12V^ by lentiviral transduction, and SW48 and SW620 cells. **b** Increased anchorage independent growth of malignant NIH3T3-KRAS^12V^ cells with addition of conditioned-media from SW620 cells that were cultured under stressed serum-free versus normal serum containing media or control growth media alone. **c** Serum-free and serum containing conditioned media from SW620 and CRC MF cells maximally induced anchorage independent growth of non-transformed NIH3T3 cells, respectively. **d** Relative cytokines levels in SW620 and CRC-MF condition media in the presence and absence of serum. **e** Secreted CXCL1 and IL8 levels in SW620 and CRC-MF conditioned media in the presence and absence of serum as measured by ELISA. Representative experiments were shown from n ≥3 experiments. P values were as indicated: *p < 0.05, **p < 0.01, ***p < 0.001.

The presence of dominant active KRAS mutation increases the ability of cells to grow under nutrient poor conditions and in an anchorage-independent manner, which is an in vitro correlate of in vivo tumorigenicity [16]. Murine NIH3T3 fiboblasts that expressed ectopic dominant active KRAS^12V^ induced a modest 3.5% efficiency in anchorage-independent growth (Figure 1a). Unmodified and ectopic GFP expressing human SW48 CRC cells, which have endogenous KRAS^WT^, had a baseline 17% soft agar growth efficiency that increased to 50% with ectopic KRAS^12V^ expression (Figure 1a). We have confirmed that human SW620 CRC cells, which have endogenous KRAS^12V^, were highly tumorigenic at 56% soft agar growth efficiency [16]. Under serum deprivation stress, SW620 cells secreted soluble factors into serum-free conditioned media that significantly enhanced soft agar growth of NIH3T3-KRAS^12V^ fibroblast in comparison to serum DMEM media control or serum containing conditioned media (Figure 1b). NIH3T3-GFP fibroblasts acquired greater anchorage-independent growth when exposed to SW620 serum-free compared to serum containing conditioned media (Figure 1c). To assess non-epithelial sources of secreted tumorigenic autocrine and paracrine factors in the tumor microenvironment, we examined CRC myofibroblasts (CRC-MF). In contrast to the effect seen with SW620 conditioned media, serum deprivation significantly decreased the tumorigenic potential of CRC-MF conditioned media on NIH3T3 fibroblasts (Figure 1c). Both human CRC epithelial cells and myofibroblasts secreted tumorigenic factors capable of inducing and enhancing anchorage-independent growth of normal and malignant NIH3T3 fibroblasts.

To identify specific tumorigenic factors present in SW620 and CRC-MF conditioned media, we screened for the relative presence of 57 cytokines, including chemokines and acute phase reactants, in the presence and absence of serum. Consistent with their potential involvement in promoting tumorigenicity of untransformed NIH3T3 and NIH3T3-KRAS^12V^ cells, only CXCL1 and IL8 levels were higher in SW620 serum-free versus serum containing conditioned media, and vice versa for CRC-MF cells (Figure 1d). Although ectopic CXCR2 expression was reported to induce NIH3T3 malignant transformation in soft agar assay [17], we detected high CXCL1 mRNA expression and very low CXCR2 mRNA expression in NIH3T3 cells (Additional file 1: Figure S1A). In comparison to SW620 conditioned media, SW48 conditioned media contained greatly reduced level of all cytokines and did not promote NIH3T3 soft agar growth (Figure 1d and data not shown). Quantitative ELISA measurement of secreted CXCL1 and IL8 levels in SW620 and CRC-MF conditioned media confirmed their maximal concentrations in the serum-free and serum containing conditioned media, respectively (Figure 1e).

### Elevated CXCL1 decreases overall survival in stage IV CRC

**Figure 2:**
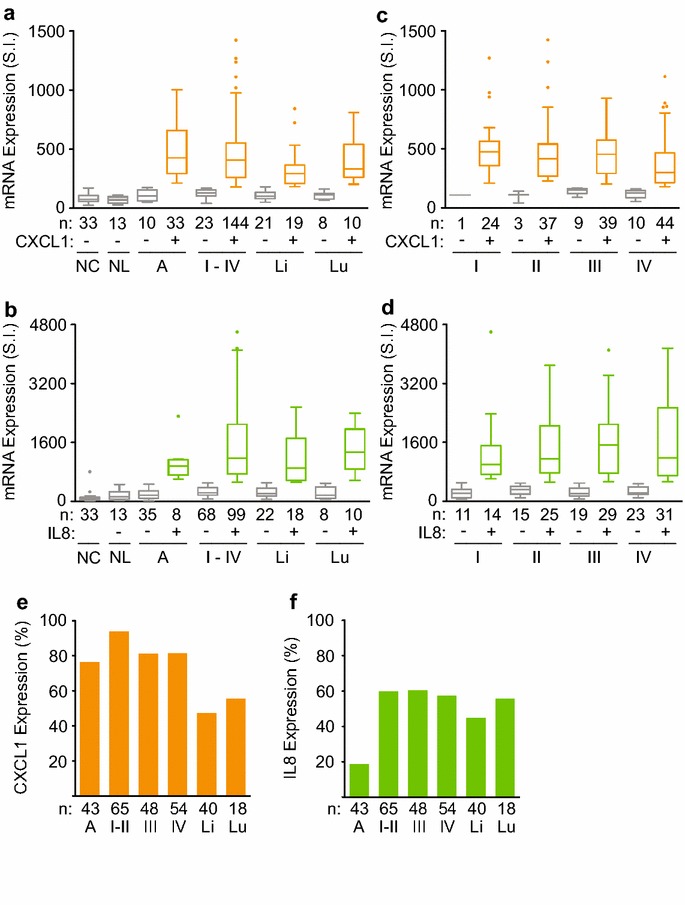
Overexpression of CXCL1 and IL8 mRNA levels in human CRC development. **a, b** Box plots of CXCL1 (**a**) and IL8 (**b**) mRNA expressions in human mucosal tissues of normal colon (NC), normal liver (NL), colon adenoma (A), stages I-IV primary colorectal adenocarcinoma (I-IV), and CRC metastases to liver (Li) and lung (Lu). **c** Box plots of CXCL1 mRNA expressions in human primary CRC stratified according to TNM stages. **d** Box plots of IL8 mRNA expressions in human primary CRC stratified according to TNM stages. **e** Percentages of human colorectal malignant tissues with elevated CXCL1 mRNA expression occurred early in most human adenoma (A) and decreased slightly with cancer metastasis. **f** Percentages of human colorectal malignant tissues with elevated IL8 mRNA expression occurred predominantly at time of conversion from adenoma to stage I–II CRC and remained elevated with disease progression. “−” and “+” indicated normal mRNA levels and those that were three standard deviations above normal, respectively. SI represented arbitrary unit of signal intensity. Patient numbers (n) were as indicated.

To determine the clinical relevance of elevated CXCL1 and IL8 levels as potential in vivo drivers of human CRC development and prognosis, we compared their RNA expression levels to overall survival. To balance out the impact of tumor and patient care heterogeneity, we analyzed human colorectal tissues from patients who were treated at a single high volume cancer center from 1991 to 2000 (Table 1). All primary stage I–IV human CRC tissues were pathologically verified. Whereas normal human tissues uniformly showed low baseline CXCL1 and IL8 levels, these levels were greater than three standard deviations above normal in most human colorectal adenoma, adenocarcinoma and metastases to the liver and lung (Figure 2a, b; Additional file 2: Table S1). Notably, elevated CXCL1 levels occurred early in 77% of colon adenomas and were sustained at high levels throughout 81–94% of primary stage I–IV CRC (Figure 2c, e). In contrast, elevated IL8 levels occurred only in 19% of colon adenomas before increasing in 57–60% of primary stage I–IV CRC (Figure 2d, f). Elevated levels of both CXCL1 and IL8 were observed in 45–56% of distant colon metastases to the liver and lung. There was a low inverse correlation between TNM stages and the mRNA levels of CXCL1 (Spearman r = −0.29; p = 0.0002) but not those of IL8 (Spearman r = 0.015; p = 0.85) (Additional file 1: Figure S1B and C).

**Table 1:** Baseline characteristics of patients

Characteristics	Adenoma	Stage I	Stage II	Stage III	Stage IV
Sex (%)					
Male	53	48	53	54	57
Female	47	52	48	46	43
Age (years)					
Median	68	68	69	66	62
Range	33–80	35–87	40–84	23–84	19–85
Tumor stage					
T status (%)					
T1 or 2	0	100	0	19	4
T3	0	0	100	75	83
T4	0	0	0	4	13
Nodal status (%)	0				
N0	0	0	0	0	30
N1	0	0	0	56	31
N2	0	0	0	44	39
Metastasis (%)	0	0	0	0	100
Type of cancer (%)					
Colon	62	68	88	81	85
Rectal	38	32	12	19	15

**Figure 3:**
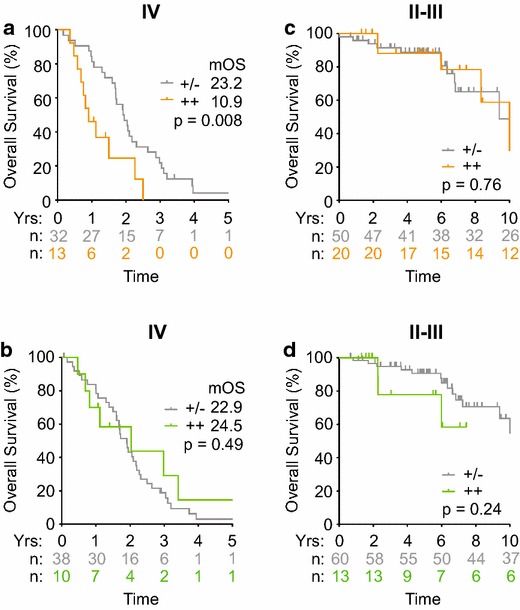
Highly elevated levels of CXCL1, but not IL8, associated with poor prognosis in stage IV human CRC. **a**–**d** Kaplan–Meier estimates of overall survival in stages IV (**a**, **b**) and II-III (**c**, **d**) CRC patients that were stratified according to normal (*gray line*; +/−), and upper-quartile (*color line*; ++) ranges of CXCL1 (**a**, **c**) or IL8 (**b**, **d**) levels. The patient numbers at risk (n) were as indicated for the groups.

To assess the prognostic significance of elevated CXCL1 and IL8, we performed Kaplan–Meier estimates of overall survival in human CRC patients. Stage IV patients with highly elevated CXCL1 levels showed a significant decrease in median overall survival of 10.9 months when compared to 23.2 months in those with normal baseline expression (Figure 3a). However, highly elevated IL8 levels in these stage IV patients did not have significant prognostic impact (Figure 3b). In our cohort of 88 stage II–III CRC patients, there was no significant overall survival difference observed between normal and elevated expression levels of CXCL1 and IL8 (Figure 3c, d). Subgroup analyses of overall survival that excluded rectal cancer patients and included only colon cancer patients, which accounted for 85% of total patients, showed similar overall survival differences (Additional file 3: Figure S2). Overall, these data indicate that CXCL1 overexpression is a poor prognostic biomarker in metastatic CRC.

### Lowering CXCL1 level decreased tumorigenic growth

**Figure 4:**
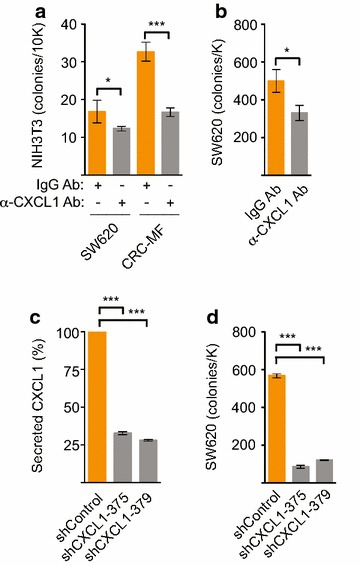
Induction of anchorage-independent growth by SW620 and CRC-MF conditioned media was dependent on CXCL1. **a** Neutralizing antibodies to CXCL1, in comparison to irrelevant isotype-specific control IgG Ab, inhibited the anchorage independent growth of non-transformed NIH3T3 cells induced by serum-free SW620 and serum-containing CRC-MF conditioned media. **b** Neutralizing antibodies to CXCL1 inhibited the anchorage independent growth of malignant SW620 cells in comparison to irrelevant isotype-specific control IgG Ab. **c**, **d** SW620 cells stably expressing two different CXCL1 specific shRNAs secreted lower CXCL1 levels (**c**) and had suppressed soft agar growth (**d**) in comparison to control shRNA. p values were as indicated: *p < 0.05, **p < 0.01, ***p < 0.001.

As elevated CXCL1 level corresponded with increased tumorigenicity induced by conditioned media and poor prognosis in stage IV CRC, we investigated the biologic and therapeutic effects of its suppression. The addition of neutralizing anti-CXCL1 antibody to SW620 and CRC-MF derived conditioned media partially inhibited their ability to induce soft agar growth of murine NIH3T3 fibroblasts (Figure 4a). Similarly, soft agar growth of highly tumorigenic SW620 cells was inhibited in the presence of neutralizing anti-CXCL1 antibody but not IgG control antibody (Figure 4b). We next generated SW620 cells that stably secreted greatly reduced CXCL1 levels in the conditioned media, as confirmed by ELISA, using two different lentiviral shRNAs targeting CXCL1 gene expression versus non-target shRNA control (Figure 4c). SW620 cells with specific reduction of secreted CXCL1 levels showed 85% and 79% reduction in soft agar colony growth (Figure 4d).

**Figure 5:**
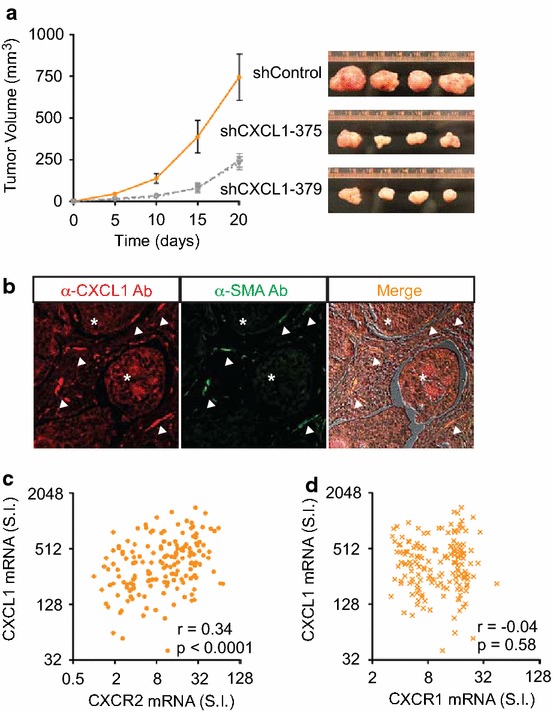
CXCL1 contributed to in vivo human CRC tumor growth. **a** SW620 cells stably expressing two distinct CXCL1 specific shRNAs had suppressed xenograft tumor growth in immunocompromised mice (n = 4 per group) with tumor images at *right*. **b** Immunofluorescence of human CRC detected CXCL1 protein expression (*red*) in CRC epithelia (*asterisks*) and co-localization with tumor myofibroblasts (*arrowheads*), which expressed α-smooth muscle actin (*green*). **c**, **d** In primary stage I–IV human CRC, mRNA levels of CXCL1 show a moderate correlation with CXCR2 (Spearman r = 0.34; CI 0.20–0.48) but not CXCR1 (Spearman r = −0.04; 95% CI −0.20 to 0.11).

To test whether CXCL1 levels correlated with in vivo tumorigenicity, we performed murine tumor xenograft assay. In comparison to those of the non-target shRNA control, the mean tumor volumes of SW620 cells were decreased by 71% and 73% in the presence of two distinct CXCL1 shRNAs (Figure 5a). We previously demonstrated CXCL1 protein expression in human CRC epithelia by immunohistochemistry [18]. Analysis of the human CRC tumor microenvironment by immunofluorescence revealed co-localization of CXCL1 protein expression with tumor myofibroblasts, which expressed α-smooth muscle actin (Figure 5b). Furthermore, we observed in primary human stage I–IV CRC a moderate correlation between CXCL1 mRNA levels and its specific CXCR2 receptor, but not CXCR1, a related chemokine receptor that does not bind CXCL1 (Figure 5c, d). We conclude that human CRC epithelia and myofibroblasts secrete elevated CXCL1 to promote in vivo tumorigenic growth.

## Discussion

While human CRC is known to involve numerous tumorigenic factors, the specific therapeutically relevant factors that significantly impact overall survival remains poorly characterized. Here, we have identified constitutively elevated levels of CXCL1 as a bona fide driver of human CRC development and poor overall survival in stage IV CRC patients. We have elucidated a complementary adaptive response to serum deprivation by human CRC epithelial cells and myofibroblasts, thereby maintaining high tumorigenic CXCL1 level in the tumor microenvironment throughout the adenoma-adenocarcinoma sequence. These findings provide new insights that indentify CXCL1 as a key targetable biomarker whose inhibition may increase overall survival in CRC patients.

Dominant active KRAS mutation is the main oncogenic driver in approximately half of sporadic metastatic colorectal adenocarcinoma [19]. In comparison to KRAS wildtype SW48 CRC epithelial cells, the KRAS mutant SW620 CRC cells used in our study demonstrated high efficiency in tumorigenic growth and secreted higher level of inflammatory chemokines such as CXCL1 and IL8. In comparison to wildtype RAS, mutant RAS also induced greater inflammatory cytokines in breast, lung, and pancreatic cancer cells [20–22]. The levels of CXCL1 and IL8 secreted are consistent with those of other human colorectal and gastric cancer cell lines [23]. We now show that the CXCL1 secreted in SW620 conditioned media are capable of inducing tumorigenic growth. Our observations that decrease CXCL1 secretion by SW620 cells inhibited their anchorage-independent and xenograft tumor growth are supported by similar findings in KRAS mutant LS174T CRC cells, whose malignant growth were inhibited by CXCL1 siRNA or neutralizing α-CXCL1 antibody [24, 25]. Treatment of KRAS mutant DLD-1 CRC cells with KRAS siRNA resulted in suppression of KRAS mutant expression and a corresponding decrease in CXCL1 level [26]. Interestingly, colorectal tumors that developed due to chronic ulcerative colitis had a lower frequency of KRAS mutation [27]. The decrease involvement of mutant KRAS in this setting may be explained by the inherently high CXCL1 expression driven by the underlying inflammatory disease [28]. Together these data suggest mutant KRAS increases inflammatory cytokines, in particular CXCL1, as a way to enhance sporadic CRC development.

Malignant tumors survive and thrive due to their great versatility in overcoming environmental limitations. Within the human CRC microenvironment, malignant epithelia and myofibroblasts drive tumor progression. Striking differences exist between the relative cellular sources of CXCL1 and IL8 secreted by epithelial cells and myofibroblasts of the human CRC microenvironment under serum enriched and deprived conditions. Consistent with their tumorigenic induction and promotion of anchorage independent growth of NIH3T3 and NIH3T3-KRAS^12V^ cells, there was a reciprocal increase and decrease in secreted CXCL1 and IL8 levels by SW620 and CRC-MF cells as a result of serum deprivation. Under optimal conditions, human CRC myofibroblasts secreted CXCL1 and IL8 at 50 and 80 fold higher levels, respectively, than human CRC epithelial cells when quantified on a per cell basis. In addition to the prominent malignant epithelia, the human CRC microenvironment had a mean stromal myofibroblasts abundance of 6% (0.4–19%) and 9% (2–24%) when assessed by α-smooth muscle actin and vimentin expressions, respectively [10, 29]. Based on mean α-smooth muscle actin expression of 6%, myofibroblasts secreted approximately threefold (range 0.2–11.7) more CXCL1 and fivefold (range 1.6–19 fold) more IL8 in comparison to malignant epithelia. This suggests that secreted CXCL1 and IL8 are derived from myofibroblasts under serum nutrient enriched condition and from human colon cancer epithelia under serum nutrient deprivation. Our finding that CXCL1 was also expressed by CRC myofibroblasts is consistent with its detection in the stroma of colonic tumor tissue microarrays [30]. In multivariate analysis of stage II–III human colon cancer patients, high expression of α-smooth muscle actin and vimentin but not stromal collagen, led to increase disease recurrence and decrease overall survival [10, 29]. Notably, a gene expression profile of human breast cancer tumor stroma that was associated with poor prognosis also showed elevated CXCL1, although the specific cell source for this secreted CXCL1 factor is difficult to determine in vivo due to potential cross contamination from breast cancer epithelia and stromal cells [8].

We extend our in vitro human cell experiments with in vivo human colorectal tissue analyses. We and others have reported that elevated levels of CXCL1 or IL8 in human colon cancer cells promotes tumorigenicity and are upregulated in human ulcerative colitis and colon cancer [18, 31–34]. We observe that elevated CXCL1 mRNA expression in human occurs throughout the adenoma-adenocarcinoma sequence with early induction in 77% of adenomas. In contrast, elevated IL8 RNA expression mainly occurs upon transition to stage I colorectal adenocarcinoma and is absent in 81% of adenomas, which is consistent with another other study [35, 36]. Our analyses showing CXCL1 overexpression in most colorectal adenomas and its low inverse correlation with TNM stages suggest that CXCL1 has important roles in both CRC initiation and tumor progression. The angiogenic and inflammatory function of CXCL1 in colorectal adenoma may contribute to new blood vessel formation and the recruitment of important supporting stromal and inflammatory cells that are critical to tumor initiation of tumor growth. We determine that the levels of CXCL1 and IL8 did not significantly affect overall survival in stage II and III human colon cancer, a finding supported by another group [37]. We also observed a significant and substantial overall survival difference in stage IV CRC that is inversely related to high expression of CXCL1 but not IL8. The lack of overall survival difference based on IL8 levels in our stage-stratified CRC patients is in contrast to a prior report that showed higher IL8 level led to decrease overall survival in non-stratified stage I–IV colon cancer patients [35, 36]. There are conflicting reports as to whether IL8 levels are associated with colorectal progression and stage [36, 37]. As both CXCL1 and IL8 are inflammatory chemokines, this suggests that the poor overall survival difference observed may be specific to CXCL1 dependent effect instead of a generalized inflammatory response.

The overall survival analysis of CXCL1 and IL8 in our study relies on comparing mRNA expression levels in human colorectal mucosal tissues to patient outcome. It is well established that both CXCL1 and IL8 production are regulated mainly at the transcriptional level by nuclear factor-κB and other transcriptional regulators [38–40]. We, along with other investigators, have validated in human colon benign and malignant tissues that CXCL1 and IL8 mRNA levels correlated with RT-PCR quantification and protein expression by immunohistochemistry [18, 34, 36, 41]. The strengths of our survival analysis in primary CRC patients included our stratification for TNM stages and pathologic confirmation of predominantly malignant tissues of moderately differentiated histology from a high volume cancer center where overall survival is better than expected with standard of care therapy. Tumor stage is the best prospectively validated prognostic indicator of overall survival in colon cancer, and tumor stage stratification is a prerequisite for prognostic biomarker analysis.

Our observation that a highly elevated level of CXCL1 is associated significantly with decreased overall survival offers an accessible extracellular target for therapeutic intervention. CXCL1 acts primarily through its receptor CXCR2. We have reported previously that the mean expression of CXCR2 was decreased in dissected mucosal tissues of human colon adenoma, primary colon adenocarcinoma and metastasis [18]. The reduction of CXCR2 level alleviates both replicative and oncogene-induced senescence and thereby promotes tumorigenesis, and in cells in that have compromised senescence machinery, such as p53 null mouse embryonic fibroblasts or NIH3T3 cells, autocrine CXCR2 signaling becomes pro-oncogenic [42]. We have observed a significant moderate correlation between levels of CXCL1 and CXCR2, suggesting that in CRC patients with high CXCL1, the CXCR2 receptor was also elevated. In a preclinical human colon cancer cell growth and metastasis model in nude mice, orally active CXCR2/CXCR1 antagonists partially decreased liver metastasis by reducing tumor neovascularization through CXCR2 signaling [43]. More effective tumor suppression may be achieved with dual CXCL1 and CXCR2 blockage.

In our study of stage IV CRC, the 12.5 months improvement in median overall survival between CXCL1 levels in the normal versus upper quartile range greatly exceeds the median overall survival improvement of 4.7 months for additional bevacizumab therapy and 3.5 months for cetuximab therapy [11, 12]. In summary, direct inhibitory targeting of CXCL1 signaling represents a very promising avenue toward overall survival benefit in human CRC.

## Conclusions

Our study suggests that highly elevated CXCL1 expression promotes tumorigenicity and serves as a poor prognostic biomarker in metastatic CRC patients. CXCL1 inhibition suppresses CRC tumor growth and warrants further investigation as a candidate therapeutic target in metastatic colorectal cancer.

## Additional files

**Additional file 1: Figure S1.** Microarray gene expressions of NIH3T3 cells and human CRC. **(A)** CXCL1, CXCR2 and DARC mRNA levels in NIH3T3 cells. **(B)** CXCL1 mRNA levels showed low inverse correlation with TNM stages of primary human CRC. **(C)** IL8 mRNA levels did not show correlation with TNM stages of primary human CRC. S.I. represented arbitrary unit of signal intensity.
**Additional file 2: Table S1.** CXCL1 and IL8 RNA expression in human colorectal cancer.
**Additional file 3: Figure S2.** Highly elevated levels of CXCL1, but not IL8, associated with poor prognosis in stage IV human colon cancer. **(A-D)** Kaplan–Meier estimates of overall survival in stages IV (A, B) and II-III (C, D) colon cancer patients that were stratified according to normal (gray line; +/−), and upper- quartile (color line; ++) ranges of CXCL1 (A, C) or IL8 (B, D) levels. The patient numbers at risk (n) were as indicated for the groups.

## Abbreviations

CRC: colorectal cancer
CXCL: chemokine (C-X-C motif) ligand
CXCR: chemokine (C-X-C motif) receptor
IL8: interleukin 8
MF: myofibroblast
