# Supplementary figures and images for: The prognostic significance of CXCL1 hypersecretion by human colorectal cancer epithelia and myofibroblasts
Source: J Transl Med. 2015 Jun 24;13:199. doi: 10.1186/s12967-015-0555-4 (PMC4477596; doi:10.1186/s12967-015-0555-4)

Figure S1

A

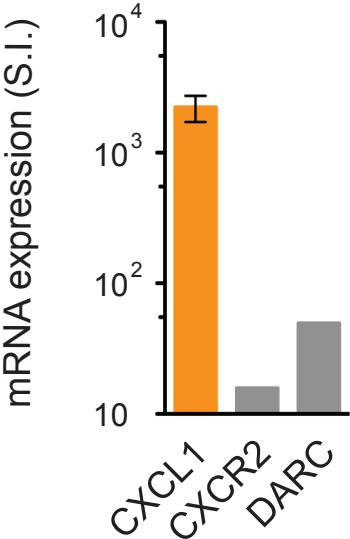

B

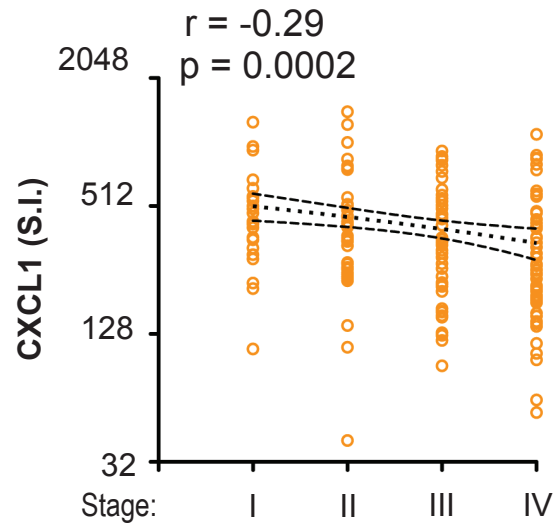

C

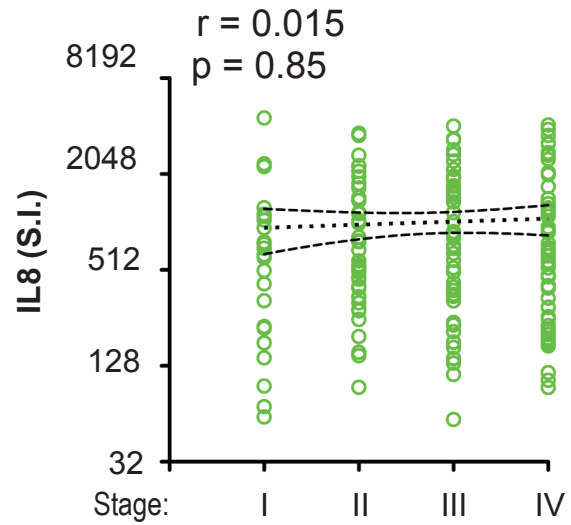

Supplement: Supplementary file 1 — Additional file 1: Figure S1. Microarray gene expressions of NIH3T3 cells and human CRC. (A) CXCL1, CXCR2 and DARC mRNA levels in NIH3T3 cells. (B) CXCL1 mRNA levels showed low inverse correlation with TNM stages of primary human CRC. (C) IL8 mRNA levels did not show correlation with TNM stages of primary human CRC. S.I. represented arbitrary unit of signal intensity. [file 12967_2015_555_MOESM1_ESM.pdf]

Figure S2

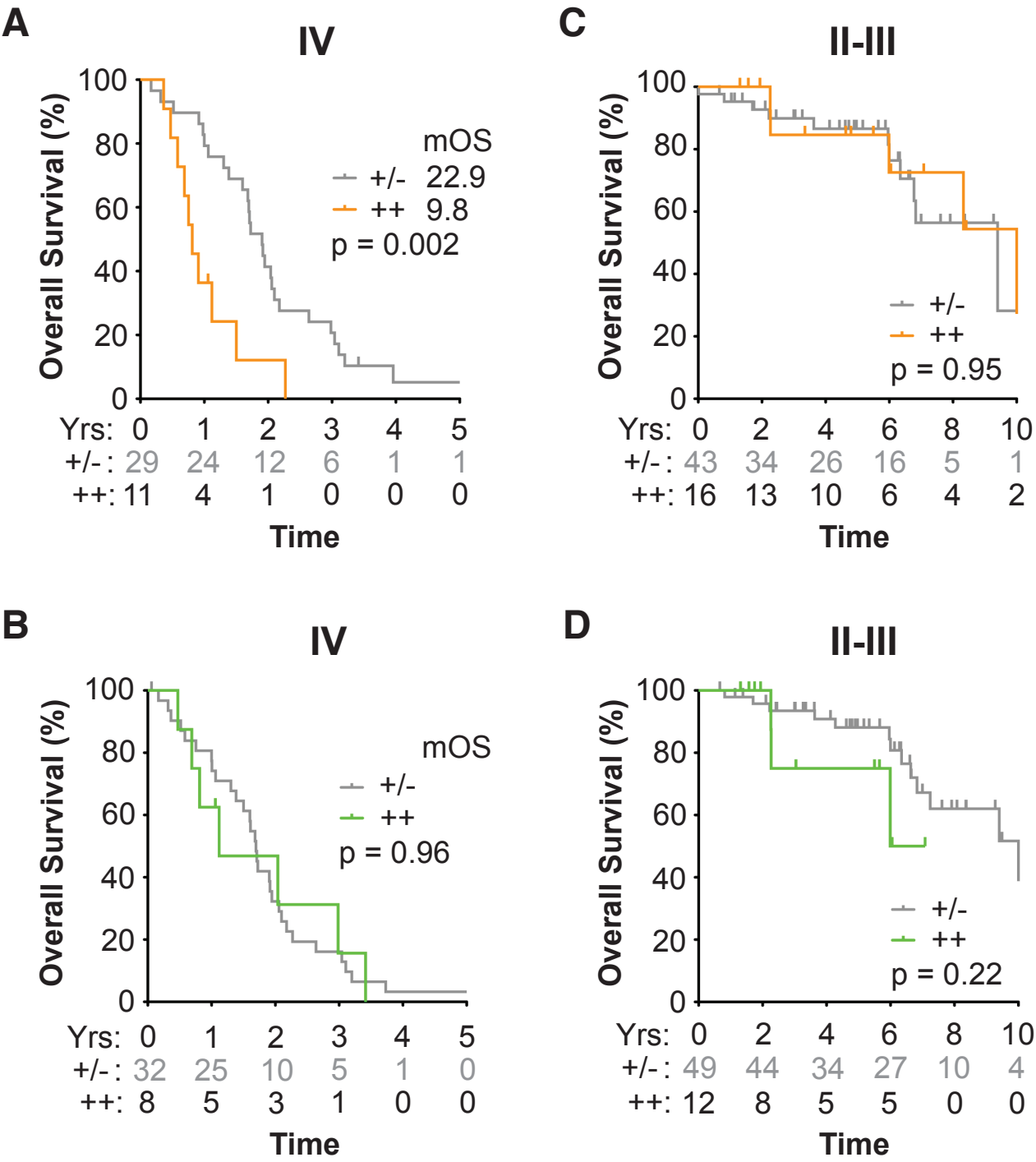

Supplement: Supplementary file 3 — Additional file 3: Figure S2. Highly elevated levels of CXCL1, but not IL8, associated with poor prognosis in stage IV human colon cancer. (A-D) Kaplan–Meier estimates of overall survival in stages IV (A, B) and II-III (C, D) colon cancer patients that were stratified according to normal (gray line; +/−), and upper- quartile (color line; ++) ranges of CXCL1 (A, C) or IL8 (B, D) levels. The patient numbers at risk (n) were as indicated for the groups. [file 12967_2015_555_MOESM3_ESM.pdf]
