# Supplementary material for: The prognostic significance of CXCL1 hypersecretion by human colorectal cancer epithelia and myofibroblasts
Source: J Transl Med. 2015 Jun 24;13:199. doi: 10.1186/s12967-015-0555-4 (PMC4477596; doi:10.1186/s12967-015-0555-4)
Supplement: Supplementary file 2 — Additional file 2: Table S1. CXCL1 and IL8 RNA expression in human colorectal cancer. [file 12967_2015_555_MOESM2_ESM.pdf]

Table S1 CXCL1 and IL8 RNA expression in human colorectal cancer

Human Tissues	CXCL1 RNA expression				IL8 RNA expression			
	Negative		Positive		Negative		Positive	
	Mean	Median	Mean	Median	Mean	Median	Mean	Median
Normal Tissue								
Colon	82 ± 33	75			98 ± 138	62		
Liver	68 ± 29	70			174 ± 135	121		
Colon Adenoma	109 ± 47	105	489± 222	426	184 ± 122	166	1062 ± 540	954
Colon Adenocarcinoma								
Stage I-II (T1-4 N0)	100 ± 42	110	501± 270	432	273 ± 135	267	1426 ± 960	1054
Stage III (T1-4 N1-2)	141 ± 26	154	467± 208	454	250 ± 123	210	1620 ± 964	1524
Stage IV (T1-4 N1-2 M1)	118 ± 38	127	387± 231	229	267 ± 119	229	1599 ± 1102	1173
Colon Metastasis								
Liver	107± 38	102	341 ± 180	295	245 ± 129	211	1174 ± 656	903
Lung	108 ± 31	111	400 ± 189	335	229 ± 168	168	1403 ± 607	1333
